# The role of transthoracic ultrasonography in predicting the outcome of community-acquired pneumonia in hospitalized children

**DOI:** 10.1371/journal.pone.0173343

**Published:** 2017-03-16

**Authors:** I-Chen Chen, Ming-Yen Lin, Yi-Ching Liu, Hsiao-Chi Cheng, Jiunn-Ren Wu, Jong-Hau Hsu, Zen-Kong Dai

**Affiliations:** 1 Department of Pediatrics, Kaohsiung Medical University Hospital, Kaohsiung, Taiwan; 2 Division of Nephrology, Department of Internal Medicine, Kaohsiung Medical University Hospital, Kaohsiung Medical University, Kaohsiung, Taiwan; 3 Faculty of Renal Care, College of Medicine, Kaohsiung Medical University, Kaohsiung, Taiwan; 4 Department of Pediatrics, School of Medicine, College of Medicine, Kaohsiung Medical University, Kaohsiung, Taiwan; University of California, Merced, UNITED STATES

## Abstract

Transthoracic ultrasound (TUS) has recently become a valuable tool in the diagnosis of community-acquired pneumonia (CAP). This study assessed the association between TUS findings and clinical outcome in children with CAP. The medical records of pediatric patients hospitalized with CAP who underwent transthoracic ultrasonography within 48 hours of admission were retrospectively reviewed. Associations between the TUS findings and patient outcome were analyzed, including intensive care unit (ICU) admission, length of hospital stay, and tube thoracotomy. The study enrolled 142 patients (median age, 60 months): 28 (19.7%) required ICU admission, 14 (9.89%) underwent tube thoracotomy, and 26 (18.3%) had a hospital stay > 9 days. Multifocal involvement seen by TUS were independently associated with ICU admission, a prolonged hospital stay, and tube thoracotomy (*p =* 0.0027, *p =* 0.02, and *p =* 0.0262, respectively). A pleural effusion and fluid bronchogram were independent predictors of a longer hospital stay (*p* = 0.003 and *p* = 0.006, respectively). In addition, a fluid bronchogram was an independent predictor of tube thoracotomy (*p* = 0.0262).

**Conclusion:**

TUS findings of fluid bronchogram, multifocal involvement, and pleural effusion were associated with adverse outcomes, including longer hospital stay, ICU admission, and tube thoracotomy in hospitalized CAP children. Therefore, TUS is a novel tool for prognostic stratifications of CAP in hospitalized children.

## Introduction

Community-acquired pneumonia (CAP) is one of the most common infectious diseases and an important cause of morbidity and mortality in children [1, 2]. Although chest radiography is currently the most commonly used tool for the detection of CAP, it is not absolutely necessary for the diagnosis in children and it involves radiation exposure [3–5]. Transthoracic ultrasound (TUS) is an emerging imaging modality in the assessment of pneumonia [6, 7]. TUS offers the advantages of more specific findings, such as an air bronchogram, fluid bronchogram, B-lines, or minimal effusion, which are often difficult to detect or differentiate by routine chest radiography. An air bronchogram reflects residual air within the consolidation, while a fluid bronchogram indicates fluid-filled airways [8]. B-lines are generated when the TUS beam is intercepted by excessive air, a liquid film, an exudate, or fibrosis in the pleural space [9]. Recent studies suggested that TUS is a feasible tool in the diagnosis and follow-up of pediatric pneumonia with the advantage of no radiation exposure [6–8, 10, 11]. However, whether TUS has a role in the prognostic stratification has not been investigated in hospitalized children with CAP.

Predicting clinical outcome of CAP can be beneficial in the management of pediatric CAP. However, to date there is no standard imaging modality for predicting the outcome of CAP. Recent studies have shown that radiographic findings are associated with the severity of CAP in children [5, 12, 13]. For example, multilobar involvement on the admission chest radiograph was related to complicated pneumonia [13, 14]. Nevertheless, the prognostic role of radiography in pediatric CAP has not been established. We postulated that TUS may have a role in predicting the clinical outcome of pediatric CAP because TUS can detect more specific and additional findings than the opacities seen on chest radiographs [8, 10, 15, 16]. Therefore, this study assessed whether TUS is valuable for predicting the outcome of CAP in hospitalized children.

## Methods

We conducted a retrospective cohort study at Kaohsiung Medical University Hospital (Kaohsiung, Taiwan). Our local Institutional Review Board approved the study (KMUHIRB-20120062). All patient records and clinical information were analyzed anonymously.

### Patients

**Fig 1:**
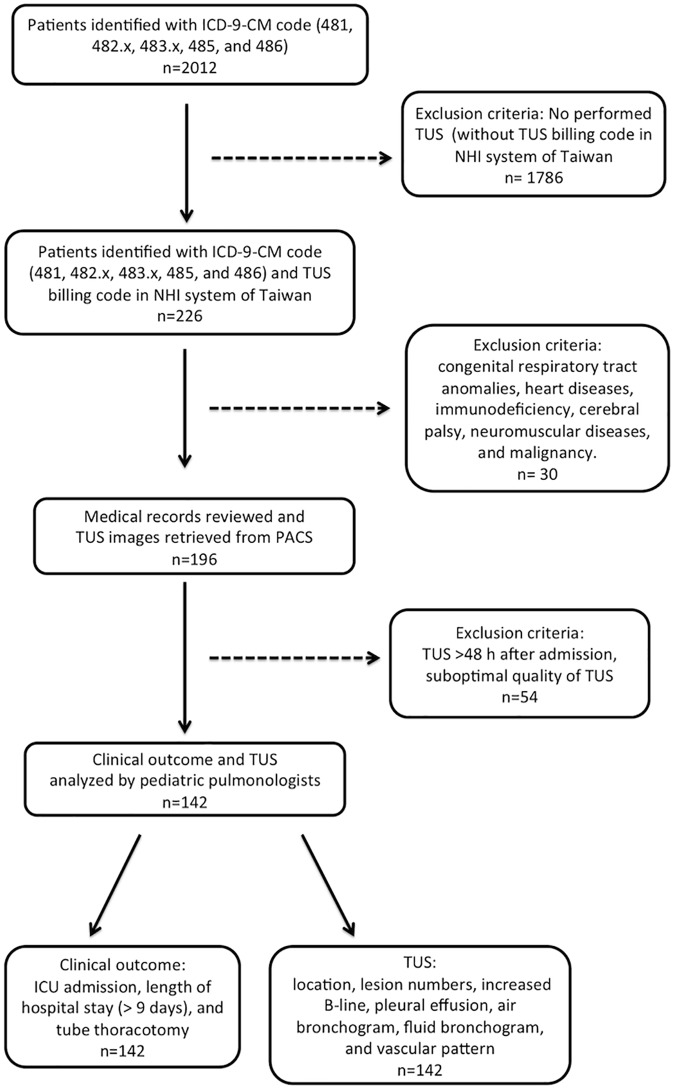
Flow-chart of the study. NHI: national health insurance; TUS: Transthoracic ultrasound; PACS: picture archiving and communication system.

We retrospectively analyzed the electric medical records of patients between 6 months and 18 years of age seen between January 1, 2010, and December 31, 2012. The inclusion criteria were children admitted to our hospital with a diagnosis of CAP and who underwent TUS within 48 hours of admission. We used International Classification of Diseases (ICD-9-CM) diagnosis codes (481, 482.x, 483.x, 485, and 486) to detect hospitalized CAP cases and the National Health Insurance billing code for TUS to identify patients for our study. (Fig 1) We excluded those who had underlying diseases, including respiratory tract anomalies, immunodeficiency, cerebral palsy, neuromuscular diseases, congenital heart disease, and malignancy. Clinical examinations were done and recorded using a digital system by a physician within 24 hours of admission. The data were extracted from our digital system of medical charts and images. Patients with poor-quality TUS were excluded from the study. Poor-quality TUS was defined as poor-resolution images or incomplete sonomorphologies. The causes of poor image quality included obesity, scoliosis and noncooperation. The diagnosis of pneumonia was made in accordance with British Thoracic Society guidelines [4].

### Clinical outcome measures

The measures of clinical outcome included the length of hospital stay, intensive care unit (ICU) admission, and complicated pneumonia requiring tube thoracotomy. To explore the factors associated with a long hospital stay, we defined a long hospital stay as > 9 days. (The 3^rd^ quartile of the length of hospitalization of all enrolled patients).

### Performance of TUS

Two pulmonologist performed these TUS scans. (I-C. C. and Z-K. D.) Location and numbers of lesions detected by TUS were recorded and all images were stored in a picture archiving and communication system. All of the enrolled patients underwent TUS within 48 hours of admission. Patients who had suboptimal or incomplete TUS were excluded. TUS was performed as described previously [10]. In brief, a TUS preset for pulmonary application with a 5.0 MHz convex probe (Sono 7500; Philips, Bothell, WA, USA) was used and interpreted by physicians with at least 3 years of experience in pediatric pulmonology.

**Fig 2:**
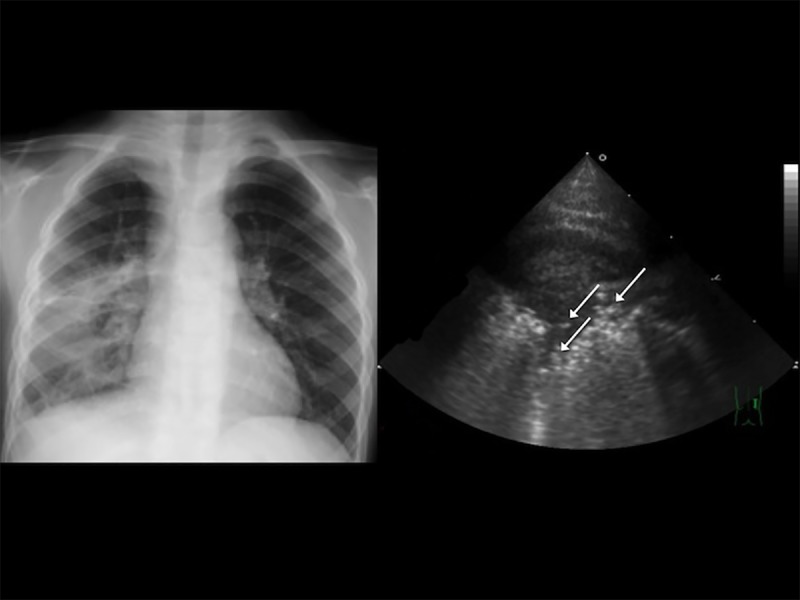
A 4-year-old boy with a high fever and dyspnea, classified as having severe pneumonia in our study. (A) Chest radiography revealed right lower lobe consolidation. (B) Transthoracic ultrasonography showed multiple hyperechoic spots in the right anterior lower lung, indicating the presence of air bronchograms (arrows).

**Fig 3:**
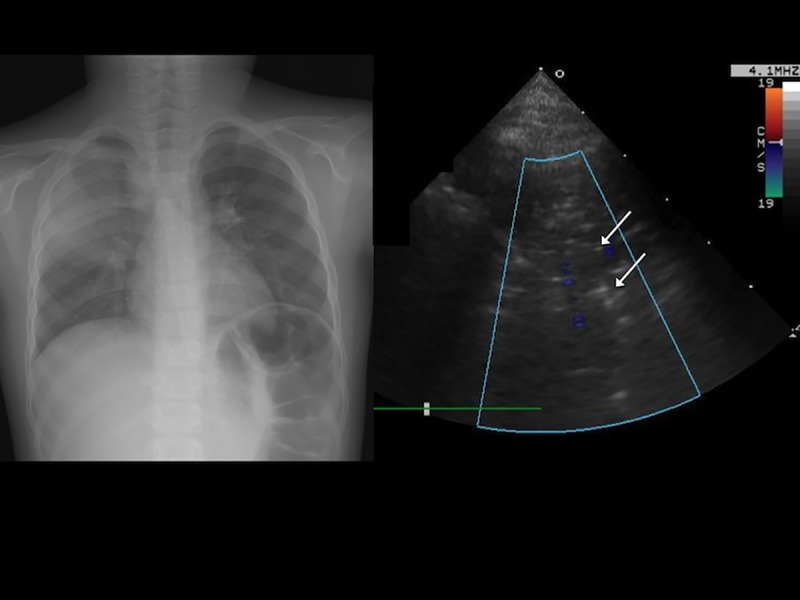
A 5-year-old boy with community-acquired pneumonia classified as severe pneumonia in our study. (A) Chest radiography revealed right upper lobe consolidation. (B) Transthoracic ultrasonography revealed multiple anechoic tubular structures without a color Doppler signal in the right anterior upper lung, indicating the presence of a fluid bronchogram (arrows).

**Fig 4:**
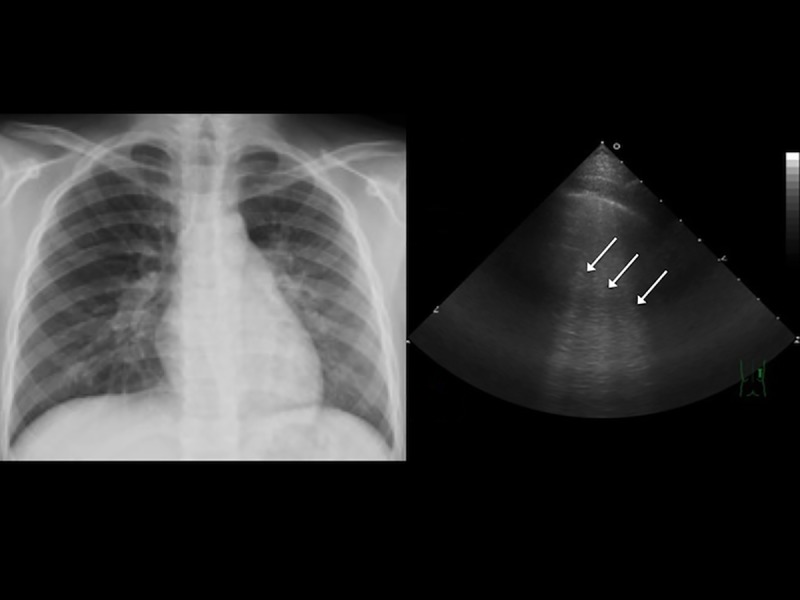
(A) Chest radiograph of a 12-year-old boy with left lower lung pneumonia. (B) Transthoracic ultrasonography revealed multiple B-lines (arrows) in the left anterior lower lung.

**Fig 5:**
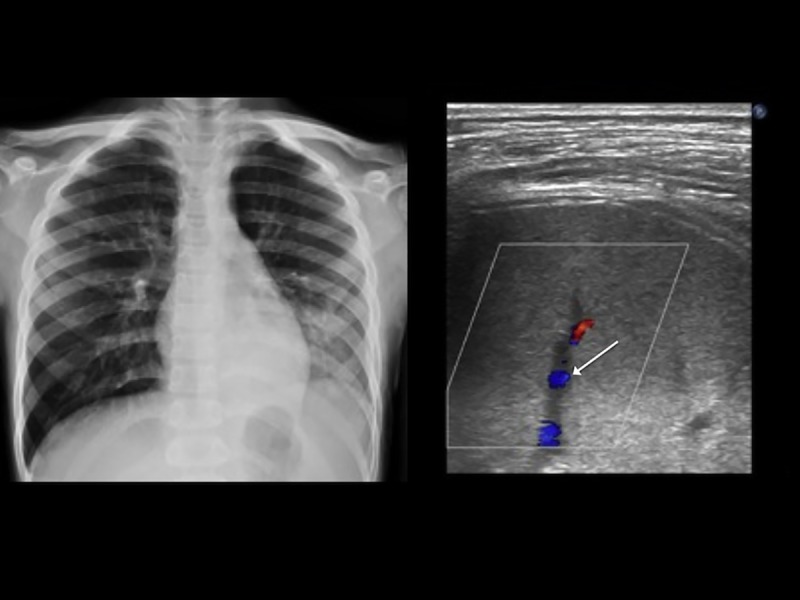
A 10-year-old boy with community acquired pneumonia. (A) Chest radiography revealed left lower lung consolidation. (B) Color Doppler sonography revealed enhanced vascularity (arrow) in the consolidation.

For each case, six sectors of each lung (upper and lower anterior, lateral, and posterior) were examined. The following TUS features of pneumonia were recorded: (1) air bronchogram (Fig 2), the presence of hyperechoic spots caused by small bubbles of trapped air; (2) fluid bronchogram (Fig 3), the presence of mucus-filled tubular structures with echogenic walls, with no color-Doppler signal; (3) increased B-lines (Fig 4), the presence of more than three B-lines per scan; (4) a vascular pattern (Fig 5), the presence of enhanced color-Doppler signal, tree-like vascularity in the consolidation; and (5) pleural effusion (6)[9, 10, 17, 18]. The focal depth of sonomorphology for detecting pneumonia in children is 5 to10 cm. We had a random sample of 10% of images to assess the inter-rater agreement.

### Data analysis

The associations and differences in continuous variables between groups of outcomes were compared using the chi-square test, Fisher’s exact test, Wilcoxon rank-sum test, and Kruskal-Wallis test, as appropriate. Univariate and multivariate analyses were used to explore the association of factors with increasing risk of severity and outcome of pneumonia based on a multiple logistic regression. These results are represented as the odds ratio (OR) with the 95% confidence interval (CI). In the multivariate analysis, all independent variables in the study were initially placed in the model and a backward selection approach was used to determine the factors significantly associated with each dependent variable. We used Kappa statistic to assess inter-rater agreement. The statistical analyses were performed using SAS 9.3 (SAS Institute, Cary, NC, USA).

## Results

### Patient characteristics

Initially, in a total of 2012 cases with ICD-9-CM diagnosis codes (481, 482.x, 483.x, 485, and 486), 226 of them have billing code of TUS in national health insurance system of Taiwan. 84 patients were excluded because they had congenital respiratory tract anomalies, heart disease, immunodeficiency, cerebral palsy, neuromuscular diseases, a malignancy, TUS > 48 hours after admission, or poor-quality TUS. The remaining 142 children were enrolled in the study. To ensure the validity of the TUS examination interpretation, we randomly selected 10% of our subjects and had an independent reviewer re-interpret the TUS examinations. The two raters had a concordance rate of 93.3% and a moderate Kappa value of 0.63, which indicated that the interpretations by the first rater were valid. Inter-observer reliability for a positive TUS with the 95% CI was defined for agreement: kappa 0.81–1.00 excellent, 0.61–0.80 good, 0.41–0.60 moderate, 0.21–0.40 fair, >0–0.20 slight, and 0 absent [19]. Table 1 summarizes the demographic, laboratory, and TUS findings of the children with CAP.

**Table 1:** Demographic data and ultrasonographic features in all 142 patients.

	Overall (n = 142)
Age, month	60 (40–96)
Male, n	74 (52%)
Body weight, kg	19 (15–26)
White blood cell count, μL^-1^	9.4 (6.7–1.6)
Outcome	
ICU admission	28 (20%)
Hospital stay, d	7.8 (5–9)
Tube thoracotomy	14 (9%)
TUS findings	
Location	
Right	82 (57%)
Left	47 (33%)
Bilateral	13 (9%)
No of lesion	
Single	114 (80%)
Multiple	28 (20%)
Increased B-lines	110 (78%)
Pleural effusion	45 (32%)
Air bronchogram	132 (93%)
Fluid bronchogram	54 (38%)
Vascular pattern	28 (20%)

ICU: intensive care unit.

TUS: Transthoracic ultrasonography.

Data are presented as median (interquartile range) or number (percentage).

### Associations between the TUS findings and clinical outcome

We examined the association between the TUS findings and clinical outcome. As shown in Table 2, multifocal involvement and pleural effusion were associated with ICU admission (*p* < 0.0001 and *p* < 0.001, respectively). In addition, multifocal involvement, increased B-lines, pleural effusion, and the presence of a fluid bronchogram were all associated with a longer hospital stay (all *p* < 0.01). Furthermore, multifocal involvement on TUS, increased B-lines, the presence of a pleural effusion, and the presence of a fluid bronchogram were all associated with tube thoracotomy (all *p* < 0.05).

**Table 2:** Association between ultrasonographic findings and clinical outcomes.

	ICU admission (n = 28)	P-value	Length of stay, d	P-value^§^	Tube thoracotomy (n = 14)	P-value	
Location		0.17		0.58^#^		0.96	
Right (n = 84)	16 (19%)		6 (5–9)		8 (9.5%)		
Left (n = 47)	7 (14%)		6 (5–8)		5 (10.6%)		
Bilateral (n = 13)	5 (38%)		8 (6–10)		1 (7.7%)		
No of lesion		<0.0001		<0.01		<0.0001	
Single (n = 114)	15 (13.2%)		6 (5–8)		5 (4.4%)		
Multiple (n = 28)	13 (46.4%)		8 (6–12)		9 (32.1%)		
B-line		0.09		<0.01		<0.05	
No (n = 32)	3 (9.4%)		5 (4–7)		0 (0.0%)		
Yes (n = 110)	25 (22.7%)		7 (5–9)		14 (12.7%)		
Pleural effusion		<0.001		<0.0001		<0.0001	
No (n = 97)	12 (12.4%)		6 (4–8)		0 (0.0%)		
Yes (n = 45)	16 (35.6%)		9 (6–12)		14 (31.1%)		
Air bronchogram		0.69		0.11		0.59	
No (n = 10)	1 (10.0%)		5 (4–7)		0 (0.0%)		
Yes (n = 132)	27 (20.5%)		6 (5–9)		14 (10.6%)		
Fluid bronchogram		0.06		<0.001		<0.001	
No (n = 88)	13 (14.8%)		6 (4–8)		4 (4.5%)		
Yes (n = 54)	15 (27.8%)		8 (5–10)		10 (18.5%)		
Vascular pattern		0.07		0.05		0.11
No (n = 114)	19 (16.7%)		6 (5–8)		9 (7.9%)		
Yes (n = 28)	9 (32.1%)		8 (5–12)		5 (17.9%)		

Data are presented as median (interquartile range) and number (percentage).

^§^ P-value was obtained using Wilcoxon rank-sum test

^#^ KrusKal-Wallis test.

### TUS findings as independent risk factors for a poor outcome

Using multivariate analyses, we examined whether the TUS findings were independent risk factors for a poor outcome and found that multifocal involvement was an independent risk factor for a poor outcome, including ICU admission (OR = 5.38), longer hospital stay (> 9 days) (OR = 9.75), and tube thoracotomy (OR = 20.12) (Table 3). In addition, the fluid bronchogram was an independent predictor of a longer hospital stay (> 9 days) (OR = 5.00) and tube thoracotomy (OR = 13.33). Pleural effusion was also an independent risk predictor of a longer hospital stay (> 9 days) (OR = 5.82). We further analyzed the association of TUS and ICU admission transferred from the general ward (n = 10). After excluding ICU patients transferred from ER (n = 18), we compared inpatients with and without ICU care (n = 10 and 114, respectively). We found that multifocal involvement was an independent risk factor for ICU admission (Odds ratio: 5.3).

**Table 3:** Significant sonomorphology for outcome (ICU admission, length of stay > 9 days, and tube thoracotomy) of pneumonia were analysis by multiple logistic regression.

Outcome	Significant sonomorphology	Odds Ratio	95% CI	P-value
ICU admission	Multifocal involvement	5.38	1.79–16.16	0.0027
> 9 days length of stay	Multifocal involvement	9.75	1.48–64.14	0.02
	Pleural effusion	5.82	1.80–18.8	0.003
	Fluid bronchogram	5.00	1.57–15.96	0.006
Tube thoracotomy	Multifocal involvement	20.12	2.97–136.41	0.0262
	Fluid bronchogram	13.33	1.36–130.87	0.0262

Abbreviation: ICU, intensive care unit; CI, confident interval.

All independent variables listed on Table 3 were included in multiple logistic regression with backward selection approach.

## Discussion

There are increasing numbers of reports on the diagnostic role of TUS in pneumonia [8, 15, 16]. However, there is little information on the role of TUS in the evaluation of the outcome of CAP in children. This study broadens the application of TUS to predicting clinical outcomes. TUS findings of multifocal involvement, a fluid bronchogram, and pleural effusion were independent predictors of a poor clinical outcome, including a prolonged hospital stay (> 9 days), ICU admission, or tube thoracotomy. To our knowledge, this is the first study demonstrating the prognostic role of TUS in pediatric patients with CAP.

Studies have shown that multifocal involvement (≥ 2 lobes or ≥ 3 lobes) revealed by chest radiography is associated with severe CAP, complicated pneumonia, acute respiratory distress syndrome, and a requirement for mechanical ventilation [7, 13, 20]. In line with these studies, we found that multifocal involvement revealed by TUS was an independent risk factor for a poor outcome (e.g., ICU stay, prolonged hospital stay, and the need for tube thoracotomy). Therefore, this study strengthens the notion that multifocal involvement, either on chest radiography or TUS, is an indicator of a poor clinical outcome for CAP patients.

Our multivariate analysis produced the novel finding that a fluid bronchogram is a risk factor for a poor outcome. A fluid bronchogram is an anechoic tubular structure along the bronchial tree and is an established feature of pneumonia that is found in approximately 20% of patients with pneumonia [10, 21]. It develops in the early phase of the disease as a result of bronchial secretions or edema [9, 21]. Since effusion is a common complication in patients with pulmonary edema [22], our findings raise the question whether a fluid bronchogram is a prelude to parapneumonic effusion due to severe or complicated pneumonia, and whether it consequently results in a worse outcome. Our results imply that once a fluid bronchogram is seen on TUS, pediatricians should be aware of the potential development of a pleural effusion and the possible need for further intervention.

TUS is a very sensitive tool for detecting parapneumonic effusions; indeed, it has been used to identify complicated pneumonia (e.g., empyema or a lung abscess). Parapneumonic effusions may exist in 36–57% of children with pneumonia [23]. In our series, 31.7% of the patients with CAP were found to have a pleural effusion by TUS at admission. Studies have shown that pleural effusions are associated with invasive pneumococcal disease [12, 13]. Intriguingly, our multivariate logistic analysis found that a pleural effusion was an independent risk factor for a prolonged hospital stay (> 9 days), but not multifocal involvement or a fluid bronchogram for tube thoracotomy. To explain this, we believe that although TUS is a very sensitive tool for detecting all pleural effusions, only a pleural effusion > 1 cm is related to a fluid bronchogram. Therefore, we re-analyzed the association between the amount of pleural effusion (> 1 cm) and a fluid bronchogram and found that a pleural effusion with a depth > 1 cm was significantly associated with a fluid bronchogram (*p* = 0.000288). This finding is in line with the guideline in the Pediatric Infectious Diseases Society and the Infectious Diseases Society of Americ}[3], suggesting >10mm and <10mm as the severity cut-off point. Further study is needed to elucidate this causal relationship.

TUS is strongly influenced by the physical interaction of the sonographic beam at the tissue/air interface, which gives rise to reverberations classified as A- or B-line (“comet tail” and “ring down”) artifacts [9]. Increased B-lines are also typical TUS findings in pneumonia. However, other conditions may also result in increased B-lines, include interstitial disease, pulmonary edema, acute bronchial asthma, and a uniformly distributed pleural effusion [24]. In a recent study, TUS is found useful to rule in pneumonia and bronchiolitis and rule out asthma in children with respiratory tract infection and wheeze [25]. Our study revealed that increased B-lines in CAP children were significantly associated with a prolonged hospital stay (*p* < 0.01) in univariate analyses. However, there was insufficient evidence supporting it as a risk factor for a poor clinical outcome in the multivariate analysis. Consequently, pediatricians should be aware that the presence of increased B-lines in pediatric CAP may reflect local inflammation around the pneumonia, and have a diagnostic but not a prognostic value [7].

The major limitation of our study is its retrospective nature and the inter-observer agreement. In addition, the sample size was too small to well characterize and analyze the full spectrum of TUS findings. Therefore, additional larger prospective studies are required to delineate the full spectrum of the relationship between TUS sonomorphology and outcome of pediatric CAP.

## Conclusion

Our study is the first to describe the association between TUS sonomorphology and clinical outcome of pediatric CAP. We found that the presence of a fluid bronchogram, pleural effusion, and multifocal involvement on TUS were risk factors for a poor clinical outcome. TUS is a potentially useful tool for predicting the outcome of CAP in hospitalized children. Further prospective studies are needed to substantiate these findings.

## Abbreviations

BTS: British Thoracic Society
CAP: community-acquired pneumonia
TUS: Transthoracic ultrasound
